# ICU stays that are judged to be non-beneficial: A qualitative study of the perception of nursing staff

**DOI:** 10.1371/journal.pone.0289954

**Published:** 2023-08-10

**Authors:** Lucas Mathey, Marine Jacquier, Nicolas Meunier-Beillard, Pascal Andreu, Jean-Baptiste Roudaut, Marie Labruyère, Jean-Philippe Rigaud, Jean-Pierre Quenot, Fiona Ecarnot

**Affiliations:** 1 Service de Médecine Intensive-Réanimation, CHU Dijon-Bourgogne, Dijon, France; 2 Equipe Lipness, Centre de Recherche INSERM UMR1231 et LabEx LipSTIC, Université de Bourgogne-Franche Comté, Dijon, France; 3 INSERM, CIC 1432, Module Épidémiologie Clinique, Université de Bourgogne-Franche Comté, Dijon, France; 4 DRCI, USMR, CHU Dijon Bourgogne, Dijon, France; 5 Department of Intensive Care, Centre Hospitalier de Dieppe, Dieppe, France; 6 Espace de Réflexion Éthique de Normandie, University Hospital Caen, Caen, France; 7 Espace de Réflexion Éthique Bourgogne Franche-Comté (EREBFC), Dijon, France; 8 EA3920, Department of Cardiology, University Hospital Besancon, Besancon, France; Harbor-UCLA Research and Education Institute: The Lundquist Institute, UNITED STATES

## Abstract

**Introduction:**

Non-beneficial stays in the intensive care unit (ICU) may have repercussions for patients and their families, but can also cause suffering among the nursing staff. We aimed explore the perceptions of nursing staff in the ICU about patient stays that are deemed to be “non-beneficial” for the patient, to identify areas amenable to intervention, with a view to improving how the nursing staff perceive the patient pathway before, during and after intensive care.

**Methods:**

Multicentre, qualitative study using individual, semi-structured interviews. All qualified nurses and nurses’ aides who were full-time employees in the ICU of three participating centres were invited to participate. Interviews were recorded, transcribed and analyzed using textual content analysis.

**Results:**

A total of 21 interviews were performed from February 2020 to October 2021, at which point saturation was reached in the data. Average age of participants was 38.5±7.5 years, and they had an average of 10.7±7.4 years of experience working in the ICU. Four major themes emerged from the interviews, namely: (1) the work is oriented towards life-threatening emergencies, technical procedures and burdensome care; (2) a range of specific criteria and circumstances influence the decisions to admit patients to ICU; (3) there are significant organisational, physical and psychological repercussions associated with a non-beneficial stay in the ICU; (4) respondents made some proposals for improvements to the patient care pathway.

**Conclusion:**

Nursing staff have a similar perception to physicians regarding admission decisions and non-beneficial ICU stays. The possibility of future ICU admission needs to be anticipated, discussed systematically with patients and integrated into healthcare goals that are consistent with the patient’s wishes and preferences, in multi-professional collaboration including nursing and medical staff.

## Introduction

The intensive care unit (ICU) is a distinct environment that occupies a central role in modern healthcare systems, due to the extensive human and technical resources deployed and the relatively small proportion of patients that benefit from this very specialized care. In the prevailing context of scarce resources, and limited availability of beds [1, 2], ICU physicians need to ensure equitable use of ICU resources, and attribution of those resources to patients in a manner that is commensurate with the patient’s healthcare goals and wishes (if and when these latter are known) [3, 4]. The right balance can be hard to find, especially in acute situations that were neither envisaged nor anticipated, and thus, were not discussed with the intensivist in advance. Such situations can lead to patients receiving care that the ICU staff ultimately deem to be “futile” or “disproportionate” [5, 6]. This can also cause suffering in the workplace for the nursing staff [7, 8], and/or cause them to lose motivation, prompting them to change units or even professions [6]. It is often difficult for physicians to estimate a patient’s prognosis when considering admission to the ICU, especially in emergency situations, where full information about the patient’s medical state may not be available, the family are not present or cannot be contacted, and the intensivist has to make a choice between a potential loss of opportunity for the patient if admission is refused, and the risk that admission may ultimately turn out to be non-beneficial for the patient [9]. Consequently, admission to the ICU of a patient whose healthcare goals are unclear, or of a patient who explicitly stated their desire NOT to be admitted to ICU, can ultimately lead the patient’s ICU stay to be considered “non-beneficial” [3, 10, 11]. The potential consequences of this include stress and anxiety among both patients and their families. Sometimes, there can even be some degree of incomprehension, which may sometimes escalate into open conflict with the healthcare team, and healthcare professionals need to be able to respond and adapt appropriately in such cases [12].

In order to anticipate such unfortunate situations and attempt to avoid their occurrence, the intensivist needs to be able to reach beyond their traditional sphere of practice, and be involved in defining the healthcare goals for patients before the occurrence of acute clinical events that may require ICU admission [13, 14]. It seems legitimate to involve ICU specialists, as they can provide expertise regarding critical care, the potential benefits of ICU admission, as well as the possible repercussions for patients and their families, in terms of physical, mental and cognitive health [15, 16].

In this study, we performed semi-structured interviews to explore the perceptions of nursing staff in the ICU about patient stays that are deemed to be “non-beneficial” for the patient. Through the prism of their experience, we sought to identify areas amenable to intervention, with a view to improving how the nursing staff perceive the patient pathway before, during and after intensive care.

## Methods

We performed a multicentre, qualitative study by means of individual, semi-structured interviews. All qualified nurses and nurses’ aides who were full-time employees in the ICU of the three participating centres (one academic teaching hospital and two general, non-academic hospitals) at the time of the study were invited to participate by personal invitation (telephone and/or email). These invitations were either sent directly, or via the hospital administration by email. Respondents were interviewed either face-to-face, or over the phone. Trainees or students were not eligible for participation. Participants were contacted by one researcher (LM; male, resident) to organize an individual semi-structured interview at a convenient time. Interviews were performed between February 2020 and October 2021 by a qualitative researcher (LM) and a sociologist with experience in clinical research and ICU care (NMB; male, PhD). There was no prior relationship between the sociologist (NMB) and the interviewees. The qualitative researcher (LM) was a medical resident in the same ICU as the interviewees, but was not hierarchically superior, thereby ruling out any potential for undue influence. To achieve maximum variation in the sample, we took account of age, total number of years’ experience since qualifying, number of years’ experience in the ICU, and the type of hospital (university teaching hospital vs non-academic general hospital).

We developed an interview guide using methods that have previously been described elsewhere [7, 17, 18]. The interview guide focused on 4 main topics, namely: (1) The mission of the ICU; (2) eligibility for ICU care (objective criteria / subjective criteria / healthcare goals / clinical status); (3) “non-beneficial” admissions; and (4) the consequences of a “non-beneficial” stay in the ICU. The interview guide was pilot tested with 3 nurses from our department; the findings from their informal interviews were not included in the analysis. The pilot interviews did not give rise to any major changes in the interview guide.

During the semi-structured interviews, the questions were open ended. The interview guide serves to prompt the respondent to talk about the aspects most important to them, in their own words. Interviews were performed in a dedicated medical office or by phone. No other persons were present during the interview. Participants were informed that quotes from their interviews might be used to substantiate the results in scientific publication, after translation into English. All participants provided written informed consent to participate in the interviews, and provided written informed consent to the use of translated quotes from their discourse in future publications. The Committee for Ethics in Research of the University of Burgundy Franche-Comté confirmed that the study did not require formal approval in compliance with French legislation.

All interviews were recorded and transcribed in their entirety for later analysis. Data were encoded to guarantee the anonymity of the participants. The discourse from the interviews was analyzed using thematic analysis as previously described by our group elsewhere [7, 17, 18]. Briefly, interviews were coded independently by 2 coauthors (LM, NMB), to identify categories, and consensually agree on themes. The different themes that arise during the interviews are noted, and classed as major themes (significant points that are of major importance and well developed by all participants) and secondary themes (less well developed by the participants). The first round of analysis was done individually by each researcher, then meetings were held to harmonize and decide on the major and secondary themes to be retained, and the optimal grouping of themes. Differences in interpretation were resolved by discussion and consensus. No software was used to assist with data management.

The analysis was validated by 4 authors (LM (reference author, male), NMB (male, sociologist, PhD), FE (female, clinical researcher, PhD), JPQ (male, critical care physician, MD, PhD). The final report was written by the same 4 authors, and approved by all. Results were not returned to the participants. Interviews were conducted in French, and translation was performed after the results were finalized. Interviews were conducted until saturation was reached (i.e. the point at which new interviews failed to bring forth any new elements on any of the points in the interview grid) [19].

## Results

A total of 28 nurses and 4 nurses’ aides were contacted, of whom 21 (65%) agreed to participate in the interview. Two nurses refused due to a lack of interest, and 9 persons did not respond despite reminders. A total of 21 interviews were performed from February 2020 to October 2021, at which point saturation was reached in the data, and thus, no further interviews were performed. The average age of participants was 38.5±7.5 years. They had an average of 14.5±8 years of experience since they qualified, and an average of 10.7±7.4 years of experience working in the ICU. The average duration of the interviews was 52.7± 12.5 minutes. The characteristics of the study population are shown in Table 1.

**Table 1:** Characteristics of the study population.

Interview number	Sex	Age	Job	Experience (years)	ICU experience (years
N°1	Female	35	Nurse’s aide	5	1.5
N°2	Male	29	Nurse	10	8
N°3	Female	28	Nurse’s aide	10	2
N°4	Male	32	Nurse	3	3
N°5	Female	29	Nurse’s aide	1	1
N°6	Female	37	Nurse	10	10
N°7	Female	34	Nurse	11	7
N°8	Female	41	Nurse	17	13
N°9	Female	47	Nurse	27	7
N°10	Female	34	Nurse	15	10
N°11	Female	39	Nurse	17	7
N°12	Female	45	Nurse	22	15
N°13	Female	32	Nurse	10	10
N°14	Male	52	Nurse’s aide	21	8
N°15	Female	46	Nurse	25	*missing*
N°16	Female	43	Nurse	20	20
N°17	Female	55	Nurse	31	31
N°18	Female	29	Nurse	5	5
N°19	Female	37	Nurse	15	10
N°20	Female	36	Nurse	13	10
N°21	Female	45	Nurse	21	20

Four major themes emerged from the interviews, namely: (1) the work is oriented towards life-threatening emergencies, technical procedures and burdensome care; (2) a range of specific criteria and circumstances influence the decisions to admit patients to ICU; (3) there are significant organisational, physical and psychological repercussions associated with a non-beneficial stay in the ICU; (4) respondents made some proposals for improvements to the patient care pathway.

Illustrative quotes for all the themes are summarized in Table 2.

**Table 2:** Illustrative quotes for each theme.

Participant ID	Quote
**Theme 1: Juggling emergencies with technical & burdensome care**	
N°15	*“There has to be monitoring of vital signs and heart rate*, *all the time”*.
N°6	*“What we do most*, *it’s obviously the technical side–managing the respirators*, *the drugs*, *dialysis…*. *That can’t be done in other units*, *because that’s really technical care”*
**Theme 2: Specific criteria/circumstances influencing admission decisions**	
N°6	*“I know they look at their previous medical history*, *and I think that’s a priority compared to age”*
N°13	*“If I see that the patient is hardly able to do anything*, *and has to have help for meals*, *or to get around*, *and stays in bed all the time*, *it’s more those criteria that bother me”*
N°9	*“When they have metastatic disease all over*, *I think it’s going overboard…*. *Wouldn’t those patients be better off with palliative care rather than intensive care*?*”*
N°19	*“More than the previous history*, *it’s the quality of life… because there are people who have adapted to their disease and live very well*, *they’re independent and living at home*, *with a good social life and activities”*
N°21	*“Take something like amyotrophic lateral sclerosis*, *diseases that have a poor quality of life*, *well……”*
N°6	*“Malnutrition*, *we all know what that causes*, *and even if we manage to deal with the respiratory problem*, *you know that the rest just won’t follow”*
N°20	*“And another thing–your patient may have to be intubated rapidly*, *and you don’t have the time to ask for consent*, *or about the prior medical history or whatever…*.*”*
N°19	*“It’s true that when you’re on night duty on your own*, *and the patient starts to deteriorate*, *then suddenly*, *the decision is basically yours to make*, *and you have decide*, *well do I intubate or not*? *Do I pursue this right through to really invasive care procedures*, *or not*?*”*
N°1	*“If you have a patient who had a cardiac arrest in another unit*, *who was resuscitated*, *with chest compressions and all*, *then they arrive here in the ICU*, *whereas in fact*, *it had already been decided that that patient should not be admitted to ICU*. *But there you go…*.*”*
N°20	*“For someone who has nothing written in their medical file about ICU admissions*, *or what to do if it happens again…*. *That’s really complicated”*
N°2	*“Youngsters who have just started don’t have enough perspective…*. *So every patient that arrives in the unit*, *they’re going to be 100% on it”*
N°13	*“Then the doctor is thinking*, *well*, *if this other doctor has called me*, *then it must serious*. *So if I don’t take the patient*, *what are they going to say*?*”*
N°14	*“If I was a doctor*, *I’d be afraid*, *just like everyone else”*
N°18	*“I think that in terms of the responsibility*, *it’s hard*, *especially when you can’t even see the patient”*
N°17	*“For some doctors*, *we know well that no matter what the patient says*, *they’ll intubate anyway*. *Because the doctor has pledged to save lives*.*”*
N°12	*“I think that some intensivists are afraid of failure*, *or in any case*, *they refuse to accept death*, *or they want to avoid the feeling that they didn’t do everything in their power*.*”*
N°2	*“Sometimes*, *it’s only for the short time needed to give the family the diagnosis*, *and for them to get used to the idea”*
N°13	*“He’s a friend of Dr*. *Whoever*, *so you kind of have to take him…*.*”*
N°18	*“It is scary to have to die*, *it puts everyone under stress*, *and it’s hard to manage*, *dealing with the family*, *the suffering*, *the pain*, *all that…”*
N°11	*“It doesn’t happen too often*, *but we have had admissions where the families were very demanding*, *and were putting pressure on the unit”*.
N°16	*“Some families can be very persuasive*, *they want you to do absolutely everything*, *to try everything”*
**Theme 3: Repercussions of stays that are later deemed “non-beneficial”**	
N°18	*“To my mind*, *we’re here to care for people*. *That’s our goal*, *but honestly*, *all the catheters*, *the blood draws…*. *And the risk of infection*! *Because in the ICU*, *even though we have very strict hygiene protocols*, *we’re generally handling a lot of bacteria that may be more or less resistant”*
N°19	*“There’s no autonomy in the ICU bedrooms*. *The quality of sleep is impaired*, *and there’s a lot more noise than in ordinary units”*
N°4	*“She finds herself in the ICU for a month*, *intubated*, *and she’s crying because she knows she’s going to die”*
N°6	*“If they’re still conscious*, *and they agreed to all this*, *then it’s because they’re afraid of dying*, *and we’re going to make them think we can help*, *when in fact*, *we can’t”*
N°11	*“[The families feel] stress*, *because it depends how they perceive the ICU*. *Some of think that ICU means*, *he’s going to die”*
N°18	*“Having a loved-one in the ICU–whether they belong there or not–it’s always very stressful for the family”*
N°9	*“If I have a lot of work with the other patients*, *then I’ll have to be quicker with the patients whose admission is not really justified; I won’t have as much time as might be necessary*.*”*
N°10	*“One thing that really annoys me is*, *when we take a patient that really shouldn’t have been admitted*, *well that leaves a niggling doubt*, *not only about your actual utility*, *but also*, *maybe we missed out on something else by taking this particular patient”*
N°10 + N°20	*“When I’m taking care of the patient*, *I feel like I’m mistreating him*, *I don’t want to do it any more*, *I’m sick of it”* *“It’s not rewarding when you’re doing a job and you honestly think that it’s pointless”*
N°10	*“I didn’t choose to work in the ICU to do palliative care”*
N°18	*“What makes me angry is when we admit patients and start treatments that I think are unsuitable*, *or that the patient had refused”*
N°9	*“I don’t feel so good*, *it bothers me*, *I put myself in the family’s position*, *or in the patient’s position*, *I would like that to happen to me”*
N°2	*“You’re not quite as involved*, *even down to the handovers*, *because you just don’t want to know everything”*
**Theme 4: Proposals for improvement to the patient pathway**	
N°16	*“We really need to ask ourselves*, *would this patient like to be in the ICU and intubated*, *or would he prefer to die at home”*
N°6	*“The family’s opinion counts as well*, *especially for older people”*
N°6	*“[The doctors] discuss it as much as they can with their colleagues*, *to decide whether or not to admit the patient to the ICU”*
N°11	*“The aim of being admitted to ICU is to deal with the acute problem*, *and enable the patient to make progress”*
N°18	*“Some patients with COPD refuse non-invasive ventilation*, *well…*. *if that means you have to intubate them*, *when in any case they’re going to refuse non-invasive ventilation again afterwards once they’re home…*. *what’s the use*?*”*
N°18	*“For someone who’s 90 years of age*, *and who’s going to end up bedridden in a nursing home…*.. *is that really worth it*?*”*
N°12	*“You could do everything for the patient*, *within the bounds of what is possible*, *and allow suitable dispositions for stopping*, *if it becomes clear that it’s not working”*
N°16	*“Communication is vital*, *and something*, *the communication is lacking*, *to help us understand this patient”*
N°4	*“Some doctors seems to think that the nurses don’t have the capacity to understand the situation”*

### 1) Perceived role of intensive care

The role of the ICU is perceived to consist in juggling life-threatening emergencies with continuous technical and burdensome care. The ICU is there to admit patients with life-threatening failure of one or multiple organ systems, and this is clearly understood by all the respondents. These emergency situations require close surveillance, as well as life-support therapies that are exclusive to the ICU, and made possible by the greater staff-to-patient ratios, which provide additional time and resources.

### 2) Specific criteria and circumstances influencing admission decisions

According to the respondents in this study, ICU physicians should systematically perform a detailed evaluation of the patient’s age, clinical history, level of autonomy and quality of life. More specifically, a recent change in the clinical status as well as the overall state of health of the patient are two parameters that the respondents emphasized as criteria that should limit eligibility for ICU admission. The respondents similarly highlighted certain diseases or clinical situations that could preclude consideration for ICU admission. However, the respondents acknowledge that in emergency or serious situations, there is not always enough time to consult the patient’s file in detail, or to consult other specialists to guide decisions.

According to our respondents, these emergency contexts too often lead to admissions that later come to be judged as non-beneficial, for patients who would later be de-prioritized for ICU access. Similarly, the failure to make decisions about non-(re)-admission of patients to ICU can render subsequent decision-making more problematic.

The caregivers interviewed in our study believe that the intensivists’ experience plays a key role in the admission procedure.

According to our respondents, some physicians find it difficult to refuse admission to the ICU because they are afraid of making the wrong decision, or being judged negatively by their peers, or because they fear legal fallout.

Furthermore, other, more individual factors can also influence the decision to admit a patient to the ICU, including some physicians’ refusal to accept death, or their profound empathy for the patient and/or family, and the fear of hurting or disappointing them.

The participants underlined the external moral pressure exerted by colleagues who insist on going as far as possible in the management of their patients, and put pressure on the intensivists to accept the patient in the ICU.

The participants also reported that families also pressure for more care, as they too refuse to see their loved one die.

### 3) Repercussions of stays that are later deemed “non-beneficial”

ICU stays that come to be deemed “non-beneficial” can lead to the delivery of care that is futile, or sometimes even harmful. The respondents reported that there is a loss of the meaning of care, related to their representations about the goals of intensive care, and this can lead to demotivation, and feelings of suffering. Furthermore, the caregivers in our study reported that they frequently encounter complaints from patients who do not comprehend the situation, and the caregivers fear giving them false hope. The same is true for the families, who report suffering from intense mental distress. Finally, with regard to “other patients”, the caregivers in our study underlined that other patients are also affected when there are non-beneficial admissions in the ICU, with a feeling among the respondents that there is inequality in the access to care.

### 4) Proposals for improvements to the patient pathway

The patients’ wishes, the family’s opinion, and collegial medical decision-making are indispensable factors to take into account with a view to improving the management of patient in the ICU, and even outside the ICU.

There is a compelling need for clearly defined healthcare goals, defined before ICU admission becomes necessary, or even after a stay in the ICU to clarify how to act in the future.

The respondents proposed the idea of a “care agreement” as a possible solution. Similar to a contract, the boundaries of care could be decided in advance of ICU admission, and in a collegial manner, to protect patients, families and caregivers against situations of deleterious therapeutic escalation.

Finally, promoting and enhancing communication between the medical and nursing teams is essential, to ensure that the goals of care for the most problematic admissions are clear to everyone, so that the nursing staff in particular can feel that their work is meaningful, and be more involved.

## Discussion

The goals of ICU admission are to offer the patient care that will not only save their life, but also guarantee an acceptable quality of life after discharge. Indeed, there are undeniable physical, mental, spiritual and financial consequences, both during and after a stay in the ICU, and affecting the patients, their families and society in general [15, 16, 20–22]. In this context, feedback should help to enhance the relevance of ICU admissions [23].

In our study, the key points that emerged from the interviews with nursing staff were as follows: first, the ICU is a distinct unit, different from other, conventional units in the hospital, because it requires more human and technical resources; second, the patient’s medical history, their healthcare pathway and healthcare goals are all important features to be taken into account when deciding on admission to the ICU; third, the intensivists’ experience on the job, their fear of making a wrong decision, moral pressure from external colleagues or from the family, refusal of death, or refusal to give up, are all factors that may modify the physicians’ perception of an ICU admission. Fourth, stress, anxiety and a failure to find meaning in their caregiving work, not to say a feeling of mistreatment or inequity regarding access to intensive care, were all mentioned as points likely to raise problems for caregivers with regard to patient stays that are “non-beneficial”. Fifth and finally, there is a compelling need to clarify healthcare goals prior to ICU admission. Developing a healthcare project that may include ICU admission requires the participation of the patient and/or family, but also, of all the caregivers in charge of the patient. This could help to limit or avoid admissions that come to be deemed “non-beneficial”, thereby also avoiding the associated consequences. Indeed, we previously reported that a lack of knowledge of the patient’s wishes, and inability to contact the family can result in ICU stays that later come to be deemed “non-beneficial” by the physicians [10]. Furthermore, non-beneficial stays may have multiple negative consequences, including stress and anxiety for the patient/family, misunderstandings and conflict with the caregiving team. In this regard, careful discussion about the possibility of future ICU admission in the framework of the patient’s overall healthcare goals has been hailed as a means to prevent non-beneficial admissions [9].

Careful reflection is a process that can undeniably improve the patient’s healthcare trajectory, as long as this reflection includes anticipation of the possible need for ICU care. Open discussion of the healthcare goals, before ICU care becomes necessary, should be systematic, and should involve the patient, the family and the caregiving team (medical and paramedical). Involvement of nurses in end-of-life discussions, a climate of trust and good relationships between nurses and physicians in the workplace, as well as intense communication have all been shown to improve the experience of nursing staff vis-à-vis end-of-life decisions and appropriateness of care. In addition, these factors also help to reduce intent-to-leave among ICU nurses [6, 8, 24]. However, some limitations to this approach have been highlighted in the literature. Firstly, physicians outside the ICU often find it difficult to accurately estimate prognosis of their patients [25–27]. Second, physicians sometimes lack sufficient perspective regarding the patient’s clinical status, prior history, potential for recovery, prior or expected future quality of life, and life story [28–30]. Third, it can be challenging to inform patients and families about potential acute deteriorations that may or may not occur [31]. Finally, there is a pervasive lack of knowledge about the specific forms of care that can be offered in the ICU [32], and likely also, a non-negligible contribution of individual emotional factors [33].

There is thus a need to rely on the knowledge of caregivers in the ICU about the consequences of an ICU stay, which depend on multiple factors, including patient characteristics such as age, comorbidities, or chronic disease [34]; frailty [35, 36]; socio-economic conditions [37, 38]; the motives for admission to ICU and the complications that occur during the ICU stay [39]. Conversely, the ICU physician, if contacted sufficiently early during the patient’s healthcare pathway, can be a key consultant (not to say the best qualified), to guide decision-making about admission (or not) to intensive care. There are several reasons for this [40]. First, the intensivist knows best what treatments (including life support therapies) can be offered in the ICU. Second, the intensivist is capable of evaluating a patient’s prognosis based on the existence or not of organ failure [24, 41]. Third, the expertise of the intensivist is essential to inform and explain to patients and their families, the detailed indications for intensive care, as well as the potential limits and consequences of ICU admission [15, 16].

In several English-speaking countries (including the UK, USA and Australia, amongst others), a process called “advance care planning” (ACP) exists, in addition to written advance directives [42]. ACP brings together the patient, the family and physicians, prior to hospitalization, in a dynamic and continuous process to define medical care that is consistent with their personal values, life goals, and preferences. It is a proactive and anticipatory approach, intended to facilitate decision-making in emergency situations, or in situations where the patient is no longer capable of clearly expressing their wishes; both often precede ICU admission. Ideally, ACP should incorporate advance directives, when they exist, but can also prompt the patient to think about writing advance directives, if none have been written. In addition to ensuring care that is in line with the patient’s preferences, numerous other benefits of ACP have been touted, especially among patients followed for chronic diseases [43–45]. It has been shown that implementing ACP increased the rate of preparation of advance directives, yielded a better balance between the care dispensed and the patient’s wishes, as well as improving communication between patients, families and healthcare professionals [4]. Nevertheless, some limitations have also been highlighted, notably the timing of ACP vis-à-vis the onset of disease, training of physicians in ACP, the difficulties for physicians to discuss death and end-of-life issues with their patients (also found in the present study), and the lack of knowledge among patients about the progressive nature of their chronic disease [44, 46].

This study was carried out during the COVID-19 pandemic, which may have modified the perceptions of the participants due to the crisis context, especially at the peak of the various waves, when ICU activity was at its most intense and stressful. We have previously reported that the pandemic led to a heavy excess burden in terms of physical work, but also in terms of mental repercussions for healthcare staff [47–51]. Indeed, healthcare workers reported psychological suffering, especially when there were organisational difficulties in the workplace, when they were working in close proximity with the patients, when they were female, and when they had to train support staff in addition to their ordinary work [47–51]. However, the unprecedented numbers of people dying during the early phases of the COVID-19 pandemic has shone a spotlight on end-of-life issues, and raised awareness among healthcare professionals and the public about the need to hold end-of-life conversations [52].

In terms of perspectives, organising pluri-professional consultations (involving physicians and nurses) after discharge of a given patient from the ICU could help to identify areas of uncertainty, and promote ethics review of the relevance of admissions (or re-admission) to ICU [53]. Similarly, the use of telemedicine could also help to better identify patients likely to benefit from intensive care [54, 55]. These avenues for future research and reflection, involving the participation of caregivers in the definition of healthcare goals, including the question of ICU admission, need to be addressed going forward. They are likely to contribute to recognizing and acknowledging the work of nursing staff, thereby improving quality of life at work, particularly the potential negative psychological impact of ICU stays that rankle, because they were “non-beneficial” [56, 57].

## Study limitations

This study has some limitations. First, as with all qualitative studies, there may be potential for desirability bias in the responses, especially when addressing questions of ethics. However, the potential for this bias was minimized by asking respondents to describe concrete examples of situations they personally experienced. Second, the interview guide may have not have encompassed the full spectrum of issues relating to non-beneficial ICU stays. We chose to limit our target study population to nurses and nurses’ aides. Consequently, the findings reported cannot be extrapolated to other professional groups working in the ICU. The number of participants may seem low, but is deemed to be sufficient since saturation was achieved [7, 17, 18, 58].

## Conclusion

Nursing staff have a similar perception to physicians regarding admission decisions and non-beneficial ICU stays. The possibility of future ICU admission needs to be anticipated, discussed systematically with patients and integrated into healthcare goals that are consistent with the patient’s wishes and preferences, in a context of pluri-professional collaboration, collegial and multidisciplinary decision-making.

## Supporting information

S1 Checklist Consolidated criteria for reporting qualitative studies (COREQ): 32-item checklist. (DOCX)

S2 Checklist Standards for Reporting Qualitative Research (SRQR)*. (DOCX)

## References

[1] Cook D, Giacomini M. The sound of silence: rationing resources for critically ill patients. Crit Care. 1999;3:R1–R3. doi: 10.1186/cc309 11094475 PMC137225

[2] Dziegielewski C, Talarico R, Imsirovic H, Qureshi D, Choudhri Y, Tanuseputro P, et al. Characteristics and resource utilization of high-cost users in the intensive care unit: a population-based cohort study. BMC Health Serv Res. 2021;21:1312. Epub 20211206. doi: 10.1186/s12913-021-07318-y 34872546 PMC8647444

[3] Nates JL, Nunnally M, Kleinpell R, Blosser S, Goldner J, Birriel B, et al. ICU Admission, Discharge, and Triage Guidelines: A Framework to Enhance Clinical Operations, Development of Institutional Policies, and Further Research. Crit Care Med. 2016;44:1553–602. doi: 10.1097/CCM.0000000000001856 27428118

[4] Quenot JP, Ecarnot F, Meunier-Beillard N, Dargent A, Large A, Andreu P, et al. What are the ethical questions raised by the integration of intensive care into advance care planning? Ann Transl Med. 2017;5:S46. doi: 10.21037/atm.2017.08.08 29302602 PMC5750251

[5] Huynh TN, Kleerup EC, Wiley JF, Savitsky TD, Guse D, Garber BJ, et al. The frequency and cost of treatment perceived to be futile in critical care. JAMA Intern Med. 2013;173:1887–94. doi: 10.1001/jamainternmed.2013.10261 24018712

[6] Piers RD, Azoulay E, Ricou B, Dekeyser Ganz F, Decruyenaere J, Max A, et al. Perceptions of appropriateness of care among European and Israeli intensive care unit nurses and physicians. JAMA. 2011;306:2694–703. doi: 10.1001/jama.2011.1888 22203538

[7] Chahraoui K, Laurent A, Bioy A, Quenot JP. Psychological experience of patients 3 months after a stay in the intensive care unit: A descriptive and qualitative study. J Crit Care. 2015;30:599–605. Epub 2015/03/18. S0883-9441(15)00079-9 [pii] doi: 10.1016/j.jcrc.2015.02.016 25776895

[8] Quenot JP, Rigaud JP, Prin S, Barbar S, Pavon A, Hamet M, et al. Suffering among carers working in critical care can be reduced by an intensive communication strategy on end-of-life practices. Intensive Care Med. 2012;38:55–61. Epub 2011/12/01. doi: 10.1007/s00134-011-2413-z 22127481 PMC3678991

[9] Quenot JP, Jacquier M, Fournel I, Meunier-Beillard N, Grange C, Ecarnot F, et al. Non-beneficial admission to the intensive care unit: A nationwide survey of practices. PLoS One. 2023;18:e0279939. Epub doi: 10.1371/journal.pone.0279939 . 36730320 PMC9894425

[10] Quenot JP, Large A, Meunier-Beillard N, Pugliesi PS, Rollet P, Toitot A, et al. What are the characteristics that lead physicians to perceive an ICU stay as non-beneficial for the patient? PLoS One. 2019;14:e0222039. doi: 10.1371/journal.pone.0222039 31490986 PMC6730882

[11] Sprung CL, Danis M, Iapichino G, Artigas A, Kesecioglu J, Moreno R, et al. Triage of intensive care patients: identifying agreement and controversy. Intensive Care Med. 2013;39:1916–24. Epub doi: 10.1007/s00134-013-3033-6 . 23925544 PMC5549951

[12] Bosslet GT, Pope TM, Rubenfeld GD, Lo B, Truog RD, Rushton CH, et al. An Official ATS/AACN/ACCP/ESICM/SCCM Policy Statement: Responding to Requests for Potentially Inappropriate Treatments in Intensive Care Units. Am J Respir Crit Care Med. 2015;191:1318–30. doi: 10.1164/rccm.201505-0924ST 25978438

[13] Quenot JP, Ecarnot F, Meunier-Beillard N, Dargent A, Large A, Andreu P, et al. What are the ethical issues in relation to the role of the family in intensive care? Ann Transl Med. 2017;5:S40. doi: 10.21037/atm.2017.04.44 29302596 PMC5750250

[14] Rigaud JP, Meunier-Beillard N, Aubry R, Dion M, Ecarnot F, Quenot JP. [The Intensive Care Physician: an External Consultant to Help Patients and Next of Kin with Decision-Making?]. Réanimation. 2016;25:367–71. 10.1007/s13546-016-1189-4.

[15] Kamdar BB, Suri R, Suchyta MR, Digrande KF, Sherwood KD, Colantuoni E, et al. Return to work after critical illness: a systematic review and meta-analysis. Thorax. 2020;75:17–27. Epub 20191108. doi: 10.1136/thoraxjnl-2019-213803 31704795 PMC7418481

[16] Needham DM, Davidson J, Cohen H, Hopkins RO, Weinert C, Wunsch H, et al. Improving long-term outcomes after discharge from intensive care unit: report from a stakeholders’ conference. Crit Care Med. 2012;40:502–9. Epub 2011/09/29. doi: 10.1097/CCM.0b013e318232da75 21946660

[17] Andreu P, Dargent A, Large A, Meunier-Beillard N, Vinault S, Leiva-Rojas U, et al. Impact of a stay in the intensive care unit on the preparation of Advance Directives: Descriptive, exploratory, qualitative study. Anaesth Crit Care Pain Med. 2018;37:113–9. doi: 10.1016/j.accpm.2017.05.007 28826983

[18] Meunier-Beillard N, Ecarnot F, Rigaud JP, Quenot JP. Can qualitative research play a role in answering ethical questions in intensive care? Ann Transl Med. 2017;5:S45. doi: 10.21037/atm.2017.09.33 29302601 PMC5750245

[19] Charmaz K. Constructing Grounded Theory: A Practical Guide through Qualitative Analysis. London: SAGE Publications Ltd.; 2006.

[20] Angus DC, Carlet J, Brussels Roundtable P. Surviving intensive care: a report from the 2002 Brussels Roundtable. Intensive Care Med. 2003;29:368–77. Epub 20030121. doi: 10.1007/s00134-002-1624-8 12536269

[21] Fernandez R, Serrano JM, Umaran I, Abizanda R, Carrillo A, Lopez-Pueyo MJ, et al. Ward mortality after ICU discharge: a multicenter validation of the Sabadell score. Intensive Care Med. 2010;36:1196–201. Epub 20100311. doi: 10.1007/s00134-010-1825-5 20221748

[22] Luce JM. End-of-life decision making in the intensive care unit. Am J Respir Crit Care Med. 2010;182:6–11. doi: 10.1164/rccm.201001-0071CI 20194809

[23] Quenot JP, Ecarnot F, Meunier-Beillard N, Dargent A, Large A, Andreu P, et al. What are the ethical aspects surrounding the collegial decisional process in limiting and withdrawing treatment in intensive care? Ann Transl Med. 2017;5:S43. doi: 10.21037/atm.2017.04.15 29302599 PMC5750242

[24] Quenot JP, Rigaud JP, Prin S, Barbar S, Pavon A, Hamet M, et al. Impact of an intensive communication strategy on end-of-life practices in the intensive care unit. Intensive Care Med. 2012;38:145–52. Epub 2011/12/01. doi: 10.1007/s00134-011-2405-z 22127479 PMC3385851

[25] Finucane TE. How gravely ill becomes dying: a key to end-of-life care. JAMA. 1999;282:1670–2. Epub 1999/11/30. jed90078 [pii]. doi: 10.1001/jama.282.17.1670 10553796

[26] Gibbins J, McCoubrie R, Alexander N, Kinzel C, Forbes K. Diagnosing dying in the acute hospital setting—are we too late? Clin Med (Lond). 2009;9:116–9. Epub 2009/05/14. doi: 10.7861/clinmedicine.9-2-116 19435113 PMC4952659

[27] Glare P, Virik K, Jones M, Hudson M, Eychmuller S, Simes J, et al. A systematic review of physicians’ survival predictions in terminally ill cancer patients. BMJ. 2003;327:195–8. Epub 2003/07/26. doi: 10.1136/bmj.327.7408.195 [doi] 327/7408/195 [pii]. 12881260 PMC166124

[28] Ferrante LE, Pisani MA, Murphy TE, Gahbauer EA, Leo-Summers LS, Gill TM. Functional trajectories among older persons before and after critical illness. JAMA Intern Med. 2015;175:523–9. Epub 2015/02/11. 2109857 [pii] doi: 10.1001/jamainternmed.2014.7889 25665067 PMC4467795

[29] Rigaud JP, Giabicani M, Beuzelin M, Marchalot A, Ecarnot F, Quenot JP. Ethical aspects of admission or non-admission to the intensive care unit. Ann Transl Med. 2017;5:S38. doi: 10.21037/atm.2017.06.53 29302594 PMC5750248

[30] Guidet B, Leblanc G, Simon T, Woimant M, Quenot JP, Ganansia O, et al. Effect of Systematic Intensive Care Unit Triage on Long-term Mortality Among Critically Ill Elderly Patients in France: A Randomized Clinical Trial. JAMA. 2017;318:1450–9. doi: 10.1001/jama.2017.13889 28973065 PMC5710364

[31] Oerlemans AJ, van Sluisveld N, van Leeuwen ES, Wollersheim H, Dekkers WJ, Zegers M. Ethical problems in intensive care unit admission and discharge decisions: a qualitative study among physicians and nurses in the Netherlands. BMC Med Ethics. 2015;16:9. Epub 20150226. doi: 10.1186/s12910-015-0001-4 25880418 PMC4344998

[32] Amin P, Fox-Robichaud A, Divatia JV, Pelosi P, Altintas D, Eryuksel E, et al. The Intensive care unit specialist: Report from the Task Force of World Federation of Societies of Intensive and Critical Care Medicine. J Crit Care. 2016;35:223–8. Epub 2016/07/23. S0883-9441(16)30121-6 [pii] doi: 10.1016/j.jcrc.2016.06.001 27444985

[33] Kozlowski D, Hutchinson M, Hurley J, Rowley J, Sutherland J. The role of emotion in clinical decision making: an integrative literature review. BMC Med Educ. 2017;17:255. Epub 20171215. doi: 10.1186/s12909-017-1089-7 29246213 PMC5732402

[34] Gayat E, Cariou A, Deye N, Vieillard-Baron A, Jaber S, Damoisel C, et al. Determinants of long-term outcome in ICU survivors: results from the FROG-ICU study. Crit Care. 2018;22:8. Epub 20180118. doi: 10.1186/s13054-017-1922-8 29347987 PMC5774139

[35] Bagshaw SM, Webb SA, Delaney A, George C, Pilcher D, Hart GK, et al. Very old patients admitted to intensive care in Australia and New Zealand: a multi-centre cohort analysis. Crit Care. 2009;13:R45. Epub 20090401. doi: 10.1186/cc7768 19335921 PMC2689489

[36] Rockwood K, Song X, MacKnight C, Bergman H, Hogan DB, McDowell I, et al. A global clinical measure of fitness and frailty in elderly people. CMAJ. 2005;173:489–95. doi: 10.1503/cmaj.050051 16129869 PMC1188185

[37] Mackenbach JP, Stirbu I, Roskam AJ, Schaap MM, Menvielle G, Leinsalu M, et al. Socioeconomic inequalities in health in 22 European countries. N Engl J Med. 2008;358:2468–81. doi: 10.1056/NEJMsa0707519 18525043

[38] Moor I, Spallek J, Richter M. Explaining socioeconomic inequalities in self-rated health: a systematic review of the relative contribution of material, psychosocial and behavioural factors. J Epidemiol Community Health. 2017;71:565–75. Epub 20160928. doi: 10.1136/jech-2016-207589 27682963

[39] Herridge MS, Tansey CM, Matte A, Tomlinson G, Diaz-Granados N, Cooper A, et al. Functional disability 5 years after acute respiratory distress syndrome. N Engl J Med. 2011;364:1293–304. doi: 10.1056/NEJMoa1011802 21470008

[40] Hilton AK, Jones D, Bellomo R. Clinical review: the role of the intensivist and the rapid response team in nosocomial end-of-life care. Crit Care. 2013;17:224. Epub 2013/05/16. cc11856 [pii] doi: 10.1186/cc11856 23672813 PMC3672544

[41] Benoit DD, Jensen HI, Malmgren J, Metaxa V, Reyners AK, Darmon M, et al. Outcome in patients perceived as receiving excessive care across different ethical climates: a prospective study in 68 intensive care units in Europe and the USA. Intensive Care Med. 2018;44:1039–49. doi: 10.1007/s00134-018-5231-8 29808345 PMC6061457

[42] Sudore RL, Lum HD, You JJ, Hanson LC, Meier DE, Pantilat SZ, et al. Defining Advance Care Planning for Adults: A Consensus Definition From a Multidisciplinary Delphi Panel. J Pain Symptom Manage. 2017;53:821–32 e1. Epub 20170103. doi: 10.1016/j.jpainsymman.2016.12.331 28062339 PMC5728651

[43] Khandelwal N, Kross EK, Engelberg RA, Coe NB, Long AC, Curtis JR. Estimating the effect of palliative care interventions and advance care planning on ICU utilization: a systematic review. Crit Care Med. 2015;43:1102–11. Epub 2015/01/13. doi: 10.1097/CCM.0000000000000852 25574794 PMC4499326

[44] Jabbarian LJ, Zwakman M, van der Heide A, Kars MC, Janssen DJA, van Delden JJ, et al. Advance care planning for patients with chronic respiratory diseases: a systematic review of preferences and practices. Thorax. 2018;73:222–30. Epub 20171106. doi: 10.1136/thoraxjnl-2016-209806 29109233

[45] Luckett T, Spencer L, Morton RL, Pollock CA, Lam L, Silvester W, et al. Advance care planning in chronic kidney disease: A survey of current practice in Australia. Nephrology (Carlton). 2017;22:139–49. doi: 10.1111/nep.12743 26860214

[46] Sedini C, Biotto M, Crespi Bel’skij LM, Moroni Grandini RE, Cesari M. Advance care planning and advance directives: an overview of the main critical issues. Aging Clin Exp Res. 2022;34:325–30. Epub 20211015. doi: 10.1007/s40520-021-02001-y 34655048 PMC8847241

[47] Laurent A, Fournier A, Lheureux F, Louis G, Nseir S, Jacq G, et al. Mental health and stress among ICU healthcare professionals in France according to intensity of the COVID-19 epidemic. Ann Intensive Care. 2021;11:90. Epub 20210604. doi: 10.1186/s13613-021-00880-y 34089117 PMC8177250

[48] Loiseau M, Ecarnot F, Meunier-Beillard N, Laurent A, Fournier A, Francois-Purssell I, et al. Mental Health Support for Hospital Staff during the COVID-19 Pandemic: Characteristics of the Services and Feedback from the Providers. Healthcare (Basel). 2022;10. Epub 20220718. doi: 10.3390/healthcare10071337 35885862 PMC9324679

[49] Ecarnot F, Lombion S, Pourrez A, Laurent A, Fournier A, Lheureux F, et al. A qualitative study of the perceptions and experiences of healthcare providers caring for critically ill patients during the first wave of the COVID-19 pandemic: A PsyCOVID-ICU substudy. PLoS One. 2022;17:e0274326. Epub 20220909. doi: 10.1371/journal.pone.0274326 36084004 PMC9462768

[50] Perraud F, Ecarnot F, Loiseau M, Laurent A, Fournier A, Lheureux F, et al. A qualitative study of reinforcement workers’ perceptions and experiences of working in intensive care during the COVID-19 pandemic: A PsyCOVID-ICU substudy. PLoS One. 2022;17:e0264287. Epub 20220304. doi: 10.1371/journal.pone.0264287 35245297 PMC8896724

[51] Laurent A, Fournier A, Lheureux F, Poujol AL, Deltour V, Ecarnot F, et al. Risk and protective factors for the possible development of post-traumatic stress disorder among intensive care professionals in France during the first peak of the COVID-19 epidemic. Eur J Psychotraumatol. 2022;13:2011603. Epub 20220126. doi: 10.1080/20008198.2021.2011603 35096285 PMC8794068

[52] Pattison N. End-of-life decisions and care in the midst of a global coronavirus (COVID-19) pandemic. Intensive Crit Care Nurs. 2020;58:102862. Epub 20200402. doi: 10.1016/j.iccn.2020.102862 32280052 PMC7132475

[53] Rigaud JP, Gelinotte S, Beuzelin M, Marchalot A, Eraldi JP, Bougerol F, et al. Post-ICU consultation: an indispensable tool in 2022. Médecine Intensive Réanimation. 2022;31:79–86. doi: 10.37051/mir-00112

[54] Fortis S, Sarrazin MV, Beck BF, Panos RJ, Reisinger HS. ICU Telemedicine Reduces Interhospital ICU Transfers in the Veterans Health Administration. Chest. 2018;154:69–76. Epub 20180615. doi: 10.1016/j.chest.2018.04.021 29914751

[55] Kalvelage C, Rademacher S, Dohmen S, Marx G, Benstoem C. Decision-Making Authority During Tele-ICU Care Reduces Mortality and Length of Stay-A Systematic Review and Meta-Analysis. Crit Care Med. 2021;49:1169–81. doi: 10.1097/CCM.0000000000004943 33710032

[56] Vanderhaeghen SFM, Decruyenaere JM, Benoit DD, Oeyen SG. Organization, feasibility and patient appreciation of a follow-up consultation in surgical critically ill patients with favorable baseline quality of life and prolonged ICU-stay: a pilot study. Acta Clin Belg. 2022:1–11. Epub 20220309. doi: 10.1080/17843286.2022.2050003 35261330

[57] Jensen JF, Thomsen T, Overgaard D, Bestle MH, Christensen D, Egerod I. Impact of follow-up consultations for ICU survivors on post-ICU syndrome: a systematic review and meta-analysis. Intensive Care Med. 2015;41:763–75. Epub 20150303. doi: 10.1007/s00134-015-3689-1 25731633

[58] Jacquier M, Meunier-Beillard N, Ecarnot F, Large A, Aptel F, Labruyere M, et al. Non-readmission decisions in the intensive care unit: A qualitative study of physicians’ experience in a multicentre French study. PLoS One. 2021;16:e0244919. Epub 20210114. doi: 10.1371/journal.pone.0244919 33444323 PMC7808577

