# Supplementary material for: ICU stays that are judged to be non-beneficial: A qualitative study of the perception of nursing staff
Source: PLoS One. 2023 Aug 10;18(8):e0289954. doi: 10.1371/journal.pone.0289954 (PMC10414562; doi:10.1371/journal.pone.0289954)
Supplement: S1 Checklist — (DOCX) [file pone.0289954.s001.docx]

**Consolidated criteria for reporting qualitative studies (COREQ): 32-item checklist**

Developed from:

Tong A, Sainsbury P, Craig J. Consolidated criteria for reporting qualitative research (COREQ): a 32-item checklist for interviews and focus groups. *International Journal for Quality in Health Care*. 2007. Volume 19, Number 6: pp. 349 – 357

**YOU MUST PROVIDE A RESPONSE FOR ALL ITEMS. ENTER N/A IF NOT APPLICABLE**

| **No. Item** | **Guide questions/description** | **Reported on Page #** |
| --- | --- | --- |
| **Domain 1: Research team and reﬂexivity** |  |  |
| *Personal Characteristics* |  |  |
| 1. Inter viewer/facilitator | Which author/s conducted the interview or focus group? | The interviews performed to develop the questionnaire were performed by NMB, the study interviews were conducted by LM and NMB. |
| 2. Credentials | What were the researcher’s credentials? E.g. PhD, MD | Sociologist PhD for Nicolas Meunier-Beillard, MD for Lucas Mathey, and MD, PhD for Jean-Pierre Quenot and Jean-Philippe Rigaud. The highest degrees for each researcher are given on the title page. |
| 3. Occupation | What was their occupation at the time of the study? | Sociologist for Nicolas Meunier-Beillard, and physicians for Lucas Mathey. |
| 4. Gender | Was the researcher male or female? | Both male, this is specified in the methods section. |
| 5. Experience and training | What experience or training did the researcher have? | The sociologist has a Masters degree in sociology, and the physicians have more than 5 years of experience in the ICU, and more than 10 years of third-level education. All had wide experience of qualitative research, as witnessed by their scientific publications (list available in Medline). This is stated in the methods.  Methods |
| *Relationship with participants* |  |  |
| 6. Relationship established | Was a relationship established prior to study commencement? | N/A |
| 7. Participant knowledge of the interviewer | What did the participants know about the researcher? e.g. personal goals, reasons for doing the research | An information letter describing the research was given to participants. Consent to participation was assumed by the fact that participation was voluntary. Ethics Committee approval was not required, in accordance with national legislation. |
| 8. Interviewer characteristics | What characteristics were reported about the inter viewer/facilitator? e.g. Bias, assumptions, reasons and interests in the research topic | The assumptions behind the development of questionnaire were based on empirical interviews as outlined in the Methods section. |
| **Domain 2: study design** |  |  |
| *Theoretical framework* |  |  |
| 9. Methodological orientation and Theory | What methodological orientation was stated to underpin the study? e.g. grounded theory, discourse analysis, ethnography, phenomenology, content analysis | Methods.  The discourse from the interviews was analyzed with thematic analysis. |
| *Participant selection* |  |  |
| 10. Sampling | How were participants selected? e.g. purposive, convenience, consecutive, snowball  . | Methods  All qualified nurses and nurses’s aides who were full-time employees in the ICU of the three participating centres (one academic teaching hospital and two general, non-academic hospitals) at the time of the study were invited to participate by personal invitation (telephone and/or email).. |
| 11. Method of approach | How were participants approached? e.g. face-to-face, telephone, mail, email | Telephone/email. |
| 12. Sample size | How many participants were in the study? | Results; 21 participants. |
| 13. Non-participation | How many people refused to participate or dropped out? Reasons? | Two nurses refused due to a lack of interest, and 9 persons did not respond despite reminders. |
| *Setting* |  |  |
| 14. Setting of data collection | Where was the data collected? e.g. home, clinic, workplace | Data collected at the hospital; Methods |
| 15. Presence of non-participants | Was anyone else present besides the participants and researchers? | No |
| 16. Description of sample | What are the important characteristics of the sample? e.g. demographic data, date | The characteristics of the respondents are given in the Results and in Table 2. |
| *Data collection* |  |  |
| 17. Interview guide | Were questions, prompts, guides provided by the authors? Was it pilot tested? | The interview guide was pilot tested with 3 nurses from our department; the findings from their informal interviews were not included in the analysis. The pilot interviews did not give rise to any major changes in the interview guide. |
| 18. Repeat interviews | Were repeat inter views carried out? If yes, how many? | No |
| 19. Audio/visual recording | Did the research use audio or visual recording to collect the data? | Audio recording (Methods) |
| 20. Field notes | Were ﬁeld notes made during and/or after the inter view or focus group? | No, since there were audio recordings |
| 21. Duration | What was the duration of the inter views or focus group? | The average duration of the interviews is given in the results and was 52.7±12.5 minutes. |
| 22. Data saturation | Was data saturation discussed? | Yes; Methods |
| 23. Transcripts returned | Were transcripts returned to participants for comment and/or correction? | No. |
| **Domain 3: analysis and ﬁndings** |  |  |
| *Data analysis* |  |  |
| 24. Number of data coders | How many data coders coded the data? | 2, with triangulation among a larger group |
| 25. Description of the coding tree | Did authors provide a description of the coding tree? | Since the interviews were confidential, this data is not freely available, but reasonable requests to the first author will be considered. |
| 26. Derivation of themes | Were themes identiﬁed in advance or derived from the data? | The themes were developed from the interviews as described in the Methods |
| 27. Software | What software, if applicable, was used to manage the data? Not applicable | None |
| 28. Participant checking | Did participants provide feedback on the ﬁndings? | No |
| *Reporting* |  |  |
| 29. Quotations presented | Were participant quotations presented to illustrate the themes/ﬁndings? Was each quotation identiﬁed? e.g. participant number | Yes, citations are given to illustrate the themes. |
| 30. Data and ﬁndings consistent | Was there consistency between the data presented and the ﬁndings? | Yes (Results). |
| 31. Clarity of major themes | Were major themes clearly presented in the ﬁndings? | Yes (Results) |
| 32. Clarity of minor themes | Is there a description of diverse cases or discussion of minor themes? | Yes Discussion |

**Once you have completed this checklist, please save a copy and upload it as part of your submission. When requested to do so as part of the upload process, please select the file type: *Checklist*. You will NOT be able to proceed with submission unless the checklist has been uploaded. Please DO NOT** **include this checklist as part of the main manuscript document. It must be uploaded as a separate file.**
